# Relatedness is a poor predictor of negative plant–soil feedbacks

**DOI:** 10.1111/nph.13238

**Published:** 2014-12-31

**Authors:** Zia Mehrabi, Sean L Tuck

**Affiliations:** 1 Long Term Ecology Laboratory, Department of Zoology, University of Oxford, OxfordOX1 3PS, UK; 2 Department of Plant Sciences, University of OxfordSouth Parks Road, Oxford, OX1 3RB, UK

**Keywords:** aboveground–belowground ecology, coexistence, Darwin's naturalization hypothesis, invasion, phylogeny, soil sickness

## Abstract

- Understanding the mechanisms underlying negative plant–soil feedbacks remains a critical challenge in plant ecology. If closely related species are more similar, then phylogeny could be used as a predictor for plant species interactions, simplifying our understanding of how plant–soil feedbacks structure plant communities, underlie invasive species dynamics, or reduce agricultural productivity.
- Here, we test the utility of phylogeny for predicting plant–soil feedbacks by undertaking a hierarchical Bayesian meta-analysis on all available pairwise plant–soil feedback experiments conducted over the last two decades, including 133 plant species in 329 pairwise interactions.
- We found that the sign and magnitude of plant–soil feedback effects were not explained by the phylogenetic distance separating interacting species. This result was consistent across different life forms, life cycles, provenances, and phylogenetic scales.
- Our analysis shows that, contrary to widespread assumption, relatedness is a poor predictor of plant–soil feedback effects.

## Introduction

**Figure 1:**
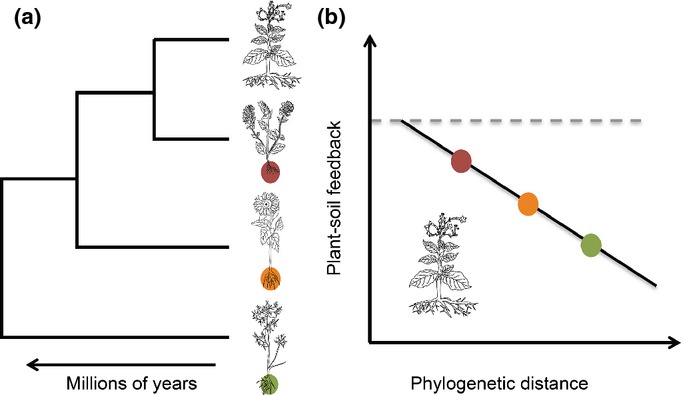
The proposed relationship between phylogeny and plant–soil feedback effects. (a) The phylogenetic relationships between species; (b) the expected outcome of the interaction between a focal species and the soils of its relatives. Negative plant–soil feedbacks represent the increased performance of species on soils cultured by heterospecifics relative to conspecifics.

Negative plant–soil feedbacks occur when conspecific adults culture soil to the relative disadvantage of their own offspring (Bever, 1994; Mangan *et al*., 2010; Van der Putten *et al*., 2013). This process can maintain diversity by generating frequency-dependent population growth, and plays key roles in agriculture and invasion ecology (Packer & Clay, 2000; Klironomos, 2002; Callaway *et al*., 2004; Petermann *et al*., 2008; Mangan *et al*., 2010). Two widely proposed mechanisms by which negative plant–soil feedbacks operate are through accumulation of host-specific soil borne pathogens and nutrient resource depletion (Kulmatiski *et al*., 2008; Mangan *et al*., 2010; Burns & Strauss, 2011; Flory & Clay, 2013; Van der Putten *et al*., 2013). Evidence that closely related plant species are more likely to share natural enemies or resources (Darwin, 1859; Webb *et al*., 2006; Gilbert & Webb, 2007) has led to a belief that plants will perform less well on soils cultured by more closely related species (Brandt *et al*., 2009; Burns & Strauss, 2011; Liu *et al*., 2012; Callaway *et al*., 2013; Sedio & Ostling, 2013) (Fig. 1). However, discontinuities between evolutionary history, plant functional traits, and niche partitioning do not support the generalizability of this hypothesis (Mouquet *et al*., 2012; Best *et al*., 2013; Narwani *et al*., 2013; Pavoine *et al*., 2013; Kelly *et al*., 2014). If traits responsible for resource use or host susceptibility to natural enemies are not widely conserved, or if the sum of plant–soil interactions remain neutral, relatedness may in fact be a poor predictor of negative plant–soil feedback effects.

More than two decades of plant–soil feedback research have generated sufficient number of studies to allow us to test the utility of evolutionary history for predicting negative plant–soil feedback effects. Here we present a formal meta-analysis of 329 experiments which compared the growth response of plant species to soil cultured by conspecifics relative to heterospecifics (133 plant species in 276 unique pairwise interactions). For each pairwise species interaction, we calculated Hedges' *d* standardized mean difference, where a negative effect size corresponds to a positive plant response to heterospecific-cultured soil relative to conspecific-cultured soil (i.e. a signal of negative plant–soil feedbacks). We then extracted the phylogenetic distances between species pairs in each experiment from a chronogram built using publicly available data. We meta-regressed our calculated effect sizes on the phylogenetic distance estimates by fitting a hierarchical Bayes linear model that accounts for sampling dependence (e.g. same conspecific control soils across multiple experiments), and hierarchical dependencies (e.g. multiple experiments within the same study) in the dataset (Stevens & Taylor, 2008). We estimated an overall slope with 95% credible intervals to test whether phylogenetic distance between species pairs could predict negative plant–soil feedbacks, and whether this relationship differed among different subsets of our dataset: plant life forms, life cycles, and native–exotic interactions.

## Materials and Methods

### Study selection

We searched all published work on ISI Web of Knowledge for the last 20 yr (1993–2013) with three topic-level searches: soil*feedback*phylogeny; soil*feedback*experiment*; and plant*feedback*soil*, and screened these alongside additional references and unpublished works for studies that recorded pairwise plant–soil feedback biomass responses. A PRISMA statement, showing this flow of information through our study selection process, can be found in Supporting Information Notes S1. All studies in our final selection had data on means and standard deviations that were readily accessible from figures or through author correspondence.

### Phylogeny estimation

Genbank accessions for five gene regions (ITS, *rbcL*, *matK*, *ndhF*, *trnL-F*) were retrieved for the species in the feedback experiments and individually aligned using MAFFT (v.7.205) with the l-ins-i algorithm (Katoh & Standley, 2013). Alignments were concatenated into a supermatrix and maximum-likelihood based estimation of the phylogeny performed using RAxML (v.7.0.4), optimized for each gene region under the GTR-GAMMA substitution model (Stamatakis, 2006). We restricted our search by using a constraint tree built from systematic treatments of the recognized tribes, families, orders and higher-level clades of the species in our study (Wojciechowski *et al*., 2006; Potter *et al*., 2007; Panero & Funk, 2008; Couvreur *et al*., 2010; Bendiksby *et al*., 2011; Soltis *et al.,* 2011; Grass Phylogeny Working Group II, 2012). We congruified (Eastman *et al*., 2013) our maximum likelihood estimate with the most resolved angiosperm chronogram currently available (Tank *et al*., 2013; Zanne *et al*., 2013), and used penalized likelihood rate smoothing to create a time scaled phylogeny of the species in our study (Smith & O'Meara, 2012). Phylogenetic distances between species in the 329 pairwise interactions were extracted using the *cophenetic.phylo* function in the R package *ape* (Paradis *et al*., 2004). Full details of the phylogeny estimation are presented in Supporting Information Methods S1.

### Hierarchical Bayesian meta-analysis

To determine the influence of phylogeny on plant–soil feedbacks we fitted a hierarchical Bayesian model accounting for sampling and hierarchical dependencies in our data. Our model in matrix form is:

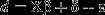
Eqn 1
where *d* is the vector of effect size estimates from all the experiments*;* X*,* a design matrix with our covariates, *β*, a vector of parameters; *δ*, a vector of hierarchical errors; and *ε* a vector of sampling errors. It assumes the distributions:

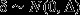


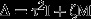


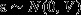
Eqn 2

where *V* is the sampling variance-covariance matrix, with known sampling variances and covariances, Δ represents the variance and covariance among random deviations of Xβ from the effect sizes being estimated. Thus, Δ is a block-diagonal matrix, with hierarchical variance τ^2^ on the diagonal (I) and blocks (M) of hierarchical covariance ζ on the off-diagonal for pairs of hierarchically dependent effect sizes. We estimated the slope for phylogenetic distance with 95% credible intervals for this estimate, calculated by multiplying the posterior standard error of the coefficients by the 95% quantile of a *t*-distribution with *N*-*k* degrees of freedom. Our analysis was carried out using R 3.1.0 (R Core Development Team, 2013) with the R package *metahdep* (Stevens & Nicholas, 2009). Further details of the methods and our study design, including the full dataset and R code used in the paper, can be found in our Supporting Information Methods S1, Notes S2 and Table S1.

## Results and Discussion

**Figure 2:**
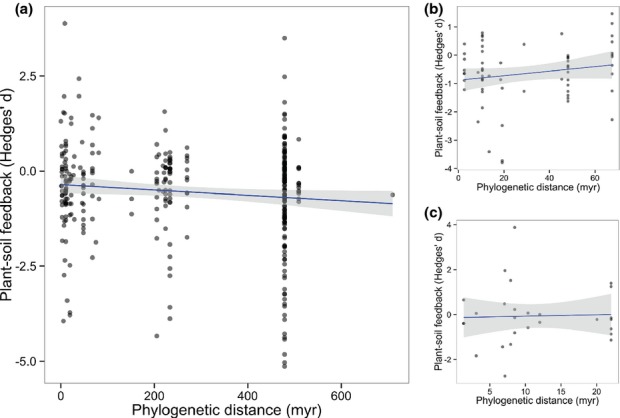
(a) Relatedness is a poor predictor of negative plant–soil feedbacks. Meta-regression of 329 experimental plant–soil feedback effects against phylogenetic distance between interacting species pairs (total species = 133). There was also no relationship between relatedness and plant-soil feedbacks for closely related species interacting within two major families in our data set, (b) Poaceae and (c) Asteraceae. Unlike the estimates shown in the text, the plotted regression slope and 95% intervals assume a fixed model with no hierarchical dependence.

Our analyses show that phylogenetic distance between species pairs is a very poor predictor of plant–soil feedback effects (−0.00035; 95% credible interval (CI): lower = −0.00077, upper = 0.00007, *n *=* *329; Fig. 2a). Whilst there was a moderately negative plant–soil feedback effect observed on average (−0.330; 95% CI: lower = −0.503, upper = −0.156, *n *=* *329), variation in magnitude of this effect could not be explained by phylogenetic distance between species pairs in plant–soil feedback interactions. As life history is linked to evolution rates and niche conservatism (Petit & Hampe, 2006; Smith & Beaulieu, 2009), we may expect heterogeneity in the response such that a phylogenetic signal in negative plant–soil feedbacks is easier to detect in longer-lived species. However, despite differences in mean feedback effects within these subgroups (i.e. intercept estimates), the lack of relationship with phylogenetic distance (i.e. slope estimates) was consistent among plant life forms (grasses, herbs, shrubs and trees) and life cycles (annual, biennial and perennial) (Table 1). It is widely argued that a phylogenetic signal exists in invasiveness (Strauss *et al*., 2006; Sol *et al*., 2014), such that we may expect distantly related species to establish and invade ecosystems through natural enemy release. However, when we analysed whether plant–soil feedback effects created in native–exotic interactions were related to phylogenetic distance between the species pairs, we found no evidence to suggest this was the case (Table 1). Finally, it has been suggested that the effect of phylogeny may be dominant between close relatives, such that relatedness might predict plant–soil feedbacks between species interacting within taxonomic divisions such as genera or tribes within plant families (e.g. Burns & Strauss, 2011; Callaway *et al*., 2013). However, we analysed plant–soil feedbacks from species interacting across a range of phylogenetic distances within two major plant families represented in our dataset (33 interactions within Asteraceae and 65 within Poaceae), and found the relationship between phylogenetic distance and plant–soil feedback interactions within these families was no different from that observed in the aggregate dataset (Poaceae: 0.0010; 95% CI: lower = −0.0079, upper = 0.0100, *n *=* *65; Asteraceae: 0.0058; 95% CI: lower = −0.0524, upper = 0.0640, *n *=* *27; Figs 2b-c). In summary, these results give little support for the notion that phylogeny can be used as a general predictor of plant–soil feedback effects.

**Table 1:** The effect of phylogeny on negative plant–soil feedbacks

	Intercept ± 95% CIs	Slope ± 95% CIs	*n*
Life form			
Grass	−0.32 ± 0.293	−0.0006 ± 0.001	124
Herb	−0.35 ± 0.422	−0.00009 ± 0.001	129
Shrub	−0.22 ± 0.572	−0.00009 ± 0.001	26
Tree	0.12 ± 0.607	−0.0004 ± 0.001	50
Life cycle			
Annual	−0.299 ± 0.451	−0.0001 ± 0.001	82
Biennial	−1.296 ± 1.374	0.0013 ± 0.004	9
Perennial	−0.224 ± 0.461	−0.0004 ± 0.001	238
Provenance			
Native	−0.207 ± 0.601	−0.0005 ± 0.002	296
Exotic	−0.853 ± 0.582	0.0017 ± 0.002	33

Intercept and slope estimates from the hierarchical Bayesian meta-analytic model. 95% credible intervals (CIs) for each estimate are calculated by multiplying the posterior standard error of the coefficients by the 95% quantile of a *t*-distribution with *N*-*k* degrees of freedom. None of the slopes showed significant effects of phylogenetic distance.

In order to check if our results were sensitive to disproportionate representation of species or phylogenetic distance levels across studies and experiments, we performed two subset analyses, re-analysing a trimmed dataset representing species in only one hierarchical grouping, and assessing the effect of unbalanced data across the range of phylogenetic distances. In order to undertake the first subset analysis with the unbiased representation of species, we trimmed the dataset to ensure species were not replicated in multiple studies, overcoming the nonindependence of multiple effect sizes for a species among studies. We selected studies for this analysis that maximized the number of total number effect sizes analysed whilst maintaining the trimming criteria. The results from this subset analysis were consistent with our initial findings: phylogenetic distance between species pairs had no effect on the magnitude of plant–soil interactions (−0.00036; 95% CI: lower = −0.00079, upper = 0.00006, *n *=* *287). The second subset analysis, for unbiased representation of phylogenetic distance, was conducted by running 100 simulations, where an effect size for each observed value on the phylogenetic distance gradient was randomly sampled, and taking the mean and standard error for this sample of slope estimates. The results from this subset analysis were also consistent with our findings that the magnitude of plant–soil feedback observed is not dependent on phylogenetic distance (mean slope = −0.00019 ± 0.00005 standard error). We checked for bias in our models using a funnel plot combined with trim and fill assessment, and a cumulative meta-analysis approach, which evaluates how the slope of the relationship between phylogenetic distance and plant–soil feedback was influenced by experiments with high sampling variance (i.e. within-study error). The model checking and subset analyses we have conducted found little evidence for publication bias in our dataset and showed that our results were robust to biases that may exist (Supporting Information Methods S1).

There is currently much debate on the use of phylogenetic data in ecology (Mayfield & Levine, 2010; Best *et al*., 2013; Narwani *et al*., 2013; Pavoine *et al*., 2013; Kelly *et al*., 2014). The growing recognition that dissimilarity in functional traits is not related in a monotonic way to phylogenetic distance suggests that phylogeny cannot be used as a general proxy for predicting the outcome of species interactions (Best *et al*., 2013; Pavoine *et al*., 2013; Kelly *et al*., 2014). The poor ability of phylogenetic distance to predict plant–soil feedbacks indicates that the traits underlying this phenomenon may be no different. Future experimental work might better focus on interactions among species at fine phylogenetic scales, and identify if and where specific traits important for plant–soil feedbacks saturate on the phylogenetic distance vs trait dissimilarity curve (e.g. Kelly *et al*., 2014). Our meta-analysis however gives strong evidence against the general use of phylogeny for predicting plant–soil feedbacks and their associated effects on plant community assembly, plant species invasions, and agricultural soil sickness.

## Supporting Information

Table S1 Study dataset

Methods S1 Supporting methods.

Notes S1 PRISMA statement.

Notes S2 R code.
