# Supplementary material for: Relatedness is a poor predictor of negative plant–soil feedbacks
Source: New Phytol. 2014 Dec 31;205(3):1071–5. doi: 10.1111/nph.13238 (PMC4303931; doi:10.1111/nph.13238)
Supplement: Methods S1 — Supporting methods. [file nph0205-1071-sd2.pdf]

Supporting Information Methods S1

Supporting Methods

Study selection. The study selection criterion was that a study presents data on the response of species grown in monoculture on soil cultured from a conspecific versus soil cultured from a heterospecific species. The response on each soil could either be biomass, survival, growth or germination. All published work on ISI Web of Knowledge for the last 20 years (Jan 1993- May 2013) were searched using 3 topic-level searches: soil* feedback* phylogeny; soil* feedback* experiment*; and plant* feedback* soil*. This returned 3560 studies, which were screened for suitability by reviewing the titles and abstracts, along with additional 68 records identified through reviews and unpublished material. After removing duplicates, 147 studies passed the initial screening and were downloaded for main text review. Out of this final selection process 41 studies were found to fit study criteria, including one study we chose to retain that reported responses of species grown on soils cultured by its own plant family versus soil cultured by a different plant family. However, not all authors provided data for these studies, and overall studies did not provide enough data to investigate responses other than biomass. As a result, our final dataset included 28 studies, comprising a sample size of 331 experiments. The analyzable number of these experiments was 329 (see supplementary R. code for details).

A PRISMA statement flowchart, showing this flow of information through our study selection process, can be found in the Supporting Information (Notes S1). All studies in our final selection had data on means and SD that were readily accessible from figures or through author correspondence. Data collection was coded after Kulmatiski

et al. (2008), but adapted to include information on plant-soil interactions between each specific plant species pair.

Calculation of phylogenetic distance. Genbank accessions for 5 gene regions (ITS, *rbcL*, *matK*, *ndhF*, *trnL-trnF*) were retrieved for the species in the feedback experiments. These five sites represent a range of both slow and fast evolving regions used for resolving phylogenetic relationships at a range of evolutionary scales. We supplemented 9 species in our study for which genetic data was not available (*Northea hornei, Pennisetum centrasiaticum, Falcataria moluccana, Ratibida pinnata, Juncus lesueurii, Juncus lesueurii, Solidago californica, Panicum sphaerocarpon, Centaurea maculosa*) with congenerics with coverage (*Northea seychellana, Cenchrus purpureus, Falcataria toona, Juncus effusus, Ratibida columnifera, Solidago canadensis, Panicum capillare, Centaurea jacea*). This substitution only reduced the phylogenetic resolution in the pairwise feedback interactions between species on one occasion (e.g. *Juncus lesueurii* vs. *Juncus lesueurii)*, and so was not problematic for our analysis. As one study in our dataset undertook a fully factorial experiment comparing six species responses on five family soils (Mehrabi, 2011), we utilized the species in the experiment as representative of the family level interactions (as all interactions in this study were across families, this did not influence the phylogenetic distance estimates and these data were not included in our analysis for testing signals within families, see Results and Discussion, Main Text). The final species coverage for gene regions were ITS (83.7%) *rbcL* (76%) *matK* (76%) *ndhF* (35.6%) and *trnL-F* (75%). Each gene was individually aligned using MAFFT (v.7.205) using the l-ins-i algorithm (Katoh & Standley, 2013). Alignments were manually checked and then concatenated into a 7009bp long supermatrix, containing a total of 60.2% missing data. Maximum-likelihood based

estimation of the phylogeny was performed using RAxML (v.7.0.4), optimized for each gene region under the general time reversible model of nucleotide substitution with gamma distributed rate heterogeneity (GTR-GAMMA) on partitioned gene regions (Stamatakis, 2006). We restricted our search by using a constraint tree built from systematic treatments of the recognized tribes, families, orders and higher-level clades of the species in our study (Wojciechowski *et al.,* 2006; Potter *et al.,* 2007; Panero & Funk, 2008; Couvreur *et al.,* 2010; Bendiksby *et al.,* 2001; Soltis *et al.,* 2011; Grass Phylogeny Working Group II, 2012). We congruified (Eastman *et al.,* 2013) our maximum likelihood estimate with the most resolved angiosperm chronogram currently available (Zanne *et al.,* 2013; Tank *et al.*, 2013), and identified 28 concordant nodes well distributed across the tree (Fig S1). For congruification we used the NCBI Taxonomy database as a reference, and manually updated a taxonomy table representing each species in the study. We then used penalized likelihood rate smoothing (optimized smoothing parameter of 0.1) to create a time scaled phylogeny of the species in our study (Smith & O'Meara, 2012). Phylogenetic distance between each species pair of the 329 pairwise interactions were extracted using the *cophenetic.phylo* function in the R package *ape* (Paradis *et al*., 2004). We checked the robustness of our results to different time scaled phylogenetic distance estimates. To do this we utilized the widely used chronogram of Bell *et al.* 2010 (with log-normal priors) as a reference tree for congruification, identified 36 congruent nodes, and time scaled our maximum likelihood estimate using penalized likelihood with node ages estimated based on upper and lower 95% HPDs for each congruent node. We then estimated the phylogenetic distances between species pairs as before. We re-ran our meta-regression models on both alternative estimates of phylogenetic distances. We found that the chronogram we employed as a time reference did not influence our

results. As a result, we present the results from phylogenetic distances estimated using the most recent chronogram in the main text (e.g. Tank *et al.* 2013).

A cumulative distribution plot of the evolutionary distances between species interacting in our study is given in Fig S2, and a chronogram used for calculating those distances is given in Fig S1. These plots show the phylogenetic distances between species interacting in the experiments in our meta-analysis covered a wide range of scales, including recent divergences (e.g. ~16% of the plant-soil feedback responses in our dataset were associated with estimated divergences of <5myrs, and ~24% with estimated divergences of <15myrs). From a taxonomic perspective, of the total plant soil feedbacks in the study, 4% were intrageneric, ~24% were intratribal and ~34% were intrafamilial.

Analysis of effect sizes. Our effect size is Hedges *d* standardised mean difference. This quantifies the plant-soil feedback effect by comparing the means of a conspecific-culture control and a heterospecific-culture treatment, standardised by the pooled standard deviation. Hedges *d* includes a small sample-size bias correction factor. A negative effect size corresponds to a positive plant response to heterospecific-cultured soil relative to conspecific-cultured soil (i.e. a signal of negative plant-soil feedback effects). The main aim of our study was not to assess the overall plant-soil feedback effect and variability in this effect among sub-groups, but instead to estimate how this effect varies with change in phylogenetic distance between the focal plant and soil-culture plant. We fitted a meta-regression to the data, estimating a slope for phylogenetic distance, to investigate whether the proposed relationship between phylogenetic distance and negative plant-soil feedback effect exists.

Our analysis was carried out using R 3.1.0 (R Core Development Team, 2013) with the R package *metahdep* (Stevens & Nicholas, 2009). The models were fitted to the data using the function *metahdep.HBLM*. We fitted these hierarchical Bayes linear models to account for sampling and hierarchical dependencies among the data. Sampling dependency exists due to multiple effect sizes using the same control group (conspecific-cultured soil). Hierarchical dependency exists among multiple effect sizes within each separate study. Defining these levels of non-independence in the data meant that a large group of extreme effect sizes within one study would be down-weighted, preventing them from having a dominant effect on the overall result. Our model in matrix form is:

$$d = X\beta + \delta + \varepsilon \qquad (1)$$

where d is the vector of effect size estimates from all the experiments; X, a design matrix with our covariates, β, a vector of parameters; δ, a vector of hierarchical errors; and ε a vector of sampling errors. It assumes the distributions:

$$\delta \sim N(0, \Delta)$$

$$\Delta = \tau^2 I + \zeta M$$

$$\varepsilon \sim N(0, V) \qquad (2)$$

Where V is the sampling variance-covariance matrix, with known sampling variances and covariances. Δ represents the variance and covariance among random deviations of Xβ from the effect sizes being estimated. Δ is a block-diagonal matrix, with hierarchical variance τ^2 on the diagonal (I) and blocks (M) of hierarchical covariance

ζ on the off-diagonal for pairs of hierarchically dependent effect sizes (Stevens and Taylor 2008). The heterogeneity between effect sizes, represented by τ^2, estimates the proportion of among-studies variance that is true variance, as opposed to within-study sampling error. This heterogeneity measure was used to estimate I^2, the proportion of total variance that is due to true variance among effect sizes ($I^2 = 0.55$) (Higgins and Thompson, 2002).

We estimated the slope for phylogenetic distance with 95% credible intervals for this estimate, calculated by multiplying the posterior standard error of the coefficients by the 95% quantile of a *t*-distribution with *N*-*k* degrees of freedom. We estimated an overall slope to quantify the relationship between phylogenetic distance and the magnitude of plant-soil feedback and then estimated slopes for individual sub-groups to assess whether this relationship varied within different families, and across different plant life forms, life cycles, and native-exotic interactions (Main Text Table 1).

Model checking. Whilst accounting for multiple levels of non-independence in our data, we were not able to account for non-independence of multiple effect sizes pertaining to the same species across studies, as opposed to within a single study. The sampling dependence of multiple within-study effect sizes that were based on the same control was accounted for, but we were unable to account for the across-study phylogenetic dependence across studies in our model framework. Instead, we performed a subset analysis, re-estimating the relationship between phylogenetic distance and plant-soil feedback after removing any species duplication across studies. This provided a conservative approach to show how robust our results were to

this species duplication.

We also assessed the presence and impact of publication bias. We used a funnel plot to assess the asymmetry in our dataset, which could suggest a greater tendency for significantly positive results to be published. To produce the funnel plot from our hierarchical model, we plotted the model residuals against precision (inverse sampling error) to account for the non-independence in the data that was dealt with in the model (Nakagawa and Santos, 2012). The model residuals calculated were conditional on the random effects (Henderson 1975). The funnel plot shows there is little evidence of publication bias that could cause skew in the distribution of residuals (Fig S3). A trim and fill analysis was used to more formally show that any bias that was present did not have any effect on the results interpretation (31 studies added, 0.0075 increase in effect size, $z = 0.2171, p = 0.8281$).

Additionally, to assess the impact of any observed bias on our results, we conducted a cumulative meta-analysis, whereby studies are cumulatively added to the analysis in the order of increasing sampling variance (Fig S4). This qualitatively shows how quickly the mean estimate stabilizes with the addition of evidence and whether the final estimate with all data included is strongly affected by the less variable studies. The cumulative meta-analysis shows that the slope estimate stabilizes quickly with increasing sample size, and that the slope estimate becomes slightly more negative with the final exclusion of effect sizes with the highest sampling variance (Fig S4).

Despite accounting for sampling and hierarchical dependencies in our dataset, as described in the methods section, a potential source of non-independence still existed among these data. In some cases, a focal species (i.e. control) occurred within multiple studies. The dependency among multiple effect sizes for a single focal species within a given study were accounted for as sampling dependency in our model. But this did not account for any dependency that may exist among multiple effect sizes for a single focal species across more than one study. To assess how sensitive our results were to this, we conducted a subset analysis, which was reported in the main text. We also reported a second subset analysis that accounted for disproportionate representation across the range of phylogenetic distance values. The methods for these subset analyses are reported in detail in the paper. Overall, these model checking procedures show that we can be confident in our estimate of the relationship between phylogenetic distance and negative plant-soil feedbacks.

References

Bell CD, Soltis DE, Soltis PS. **2010**. The age and diversification of the angiosperms revisited. *American Journal of Botany.* **97**: 1296–1303.

Bendiksby M, Thorbek L, Scheen A, Lindwvist C,& Ryding O. 2011. An updated phylogeny and classification of Lamiaceae subfamily Lamiodeae. *Taxon.* **60:** 471-484

Couvreur TLP, Franzke A, Al-shehbaz IA, Bakker FT, Koch A, Mummenhoff K. **2010**. Molecular Phylogenetics , Temporal Diversification , and Principles of Evolution in the Mustard Family (Brassicaceae). *Molecular Biology and Evolution.* **27**: 55–71.

Eastman JM, Harmon LJ, Tank DC. **2013**. Congruification : support for time scaling large phylogenetic trees. *Methods in Ecology and Evolution.* **4**: 688–691.

Grass Phylogeny Working Group 2012. New grass phylogeny resolves deep evolutionary relationships and discovers C4 origins. *New Phytologist*: **193**: 304–312.

Katoh K, Standley DM. **2013**. MAFFT Multiple Sequence Alignment Software Version 7 : Improvements in Performance and Usability Article Fast Track. *Molecular Biology and Evolution.* **30**: 772–780.

Henderson C. **1975**. Best linear unbiased estimation and prediction under a selection model. *Biometrics*. **31**: 423

Higgins JPT, Thompson SG. **2002**. Quantifying heterogeneity in a meta-analysis. *Statistics in medicine* **21**: 1539–58.

Mehrabi Z. 2011. Close relatives don't make bad nieghbours: plant soil feedbacks and the dynamics of plant communities. Undergradute Thesis. University of Oxford.

Nakagawa S, Santos ES 2012. Methodological issues and advances in biological meta-analysis. *Evolutionary Ecology* **26**: 1253–1274.

Paradis E., Claude J. & Strimmer K. 2004. APE: analyses of phylogenetics and evolution in R language. *Bioinformatics* **20**: 289-290.

Panero JL, Funk VA. **2008**. The value of sampling anomalous taxa in phylogenetic studies : Major clades of the Asteraceae revealed. *Molecular Phylogenetics and Evolution.* **47**: 757–782.

Potter D, Eriksson T, Evans RC, Oh S, Smedmark JEE, Morgan DR, Kerr M. **2007**. Evolution Phylogeny and classification of Rosaceae. *Plant systematics and evolution.* **266:** 5-43.

R Core Development Team. **2013**. *R: a language and environment for statistical computing v.3.0.1* (R Foundation for Statistical Computing, 2013)

Smith SA, O'Meara BCO. **2012**. treePL : divergence time estimation using penalized likelihood for large phylogenies. *Bioinformatics.* **28**: 2689–2690.

Soltis DE, Smith SA, Cellinese N, Wurdack KJ, Tank DC, Brockington SF, Refulio-rodriguez NF, Walker JB, Moore MJ, Carlsward BS, *et al.* 2011. Angiosperm phylogeny: 17 genes, 640 taxa. *American Journal of Botany.***98**: 704–730.

Stamatakis A. **2006**. RAxML-VI-HPC : maximum likelihood-based phylogenetic analyses with thousands of taxa and mixed models. *Bioinformatics.* **22**: 2688–2690.

Stevens JR, Nicholas G. **2009**. Metahdep: Meta-Analysis of Hierarchically Dependent Gene Expression Studies. *Bioinformatics* **25**: 2619–2620.

Stevens JR, Taylor M. **2008**. Hierarchical Dependence in Meta-Analysis. *Journal of Educational and Behavioral Statistics* **34**: 46–73.

Tank, D. C., Eastman, J. M., Beaulieu, J. M. & Smith, S. A. 2013. Phylogenetic resources. Data from: Three keys to the radiation of angiosperms into freezing environments. Nature. Dryad Digital Repository. http://dx.doi.org/10.5061/dryad.63q27/3.

Wojciechowski, Martin F., Johanna Mahn, and Bruce Jones. 2006. Fabaceae. legumes. [Online]: http://tolweb.org/Fabaceae/21093/2006.06.14 *in* The Tree of Life Web Project, http://tolweb.org/ on 25th October 2014

Zanne AE, Tank DC, Cornwell WK, Eastman JM, Smith SA, FitzJohn RG, McGlinn DJ, O'Meara BC, Moles AT, Reich PB, Royer DL, *et al.* 2013. Three keys to the radiation of angiosperms into freezing environments. *Nature.* **506**: 89–92.

Supplementary figures

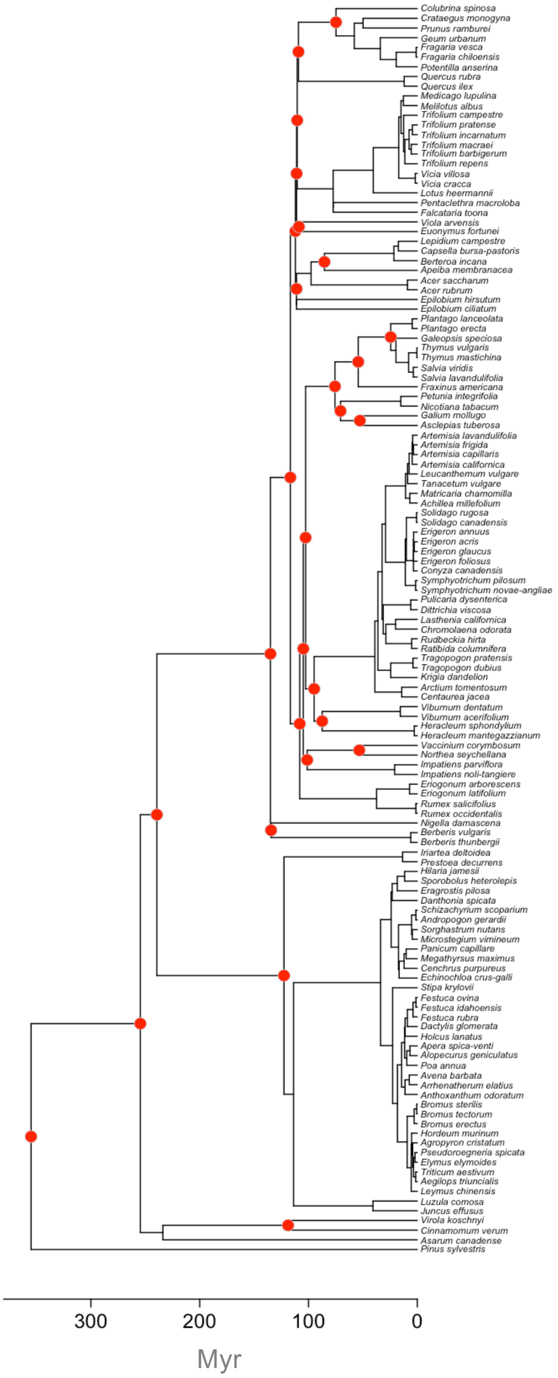

Fig S1 Dated maximum likelihood estimate of the phylogenetic relationships between the species used in the meta-analysis. Red dots represent the congruent nodes with the Tank *et al*. 2013 chronogram used for time scaling the estimate.

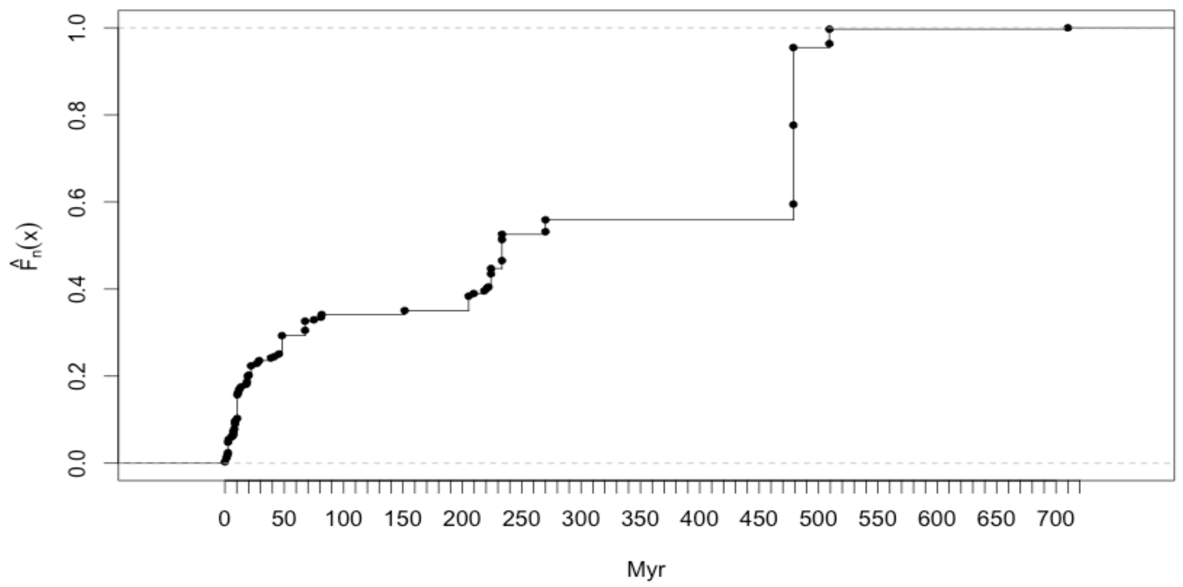

Fig S2 Cumulative distribution of the phylogenetic distances between species interacting in our study.

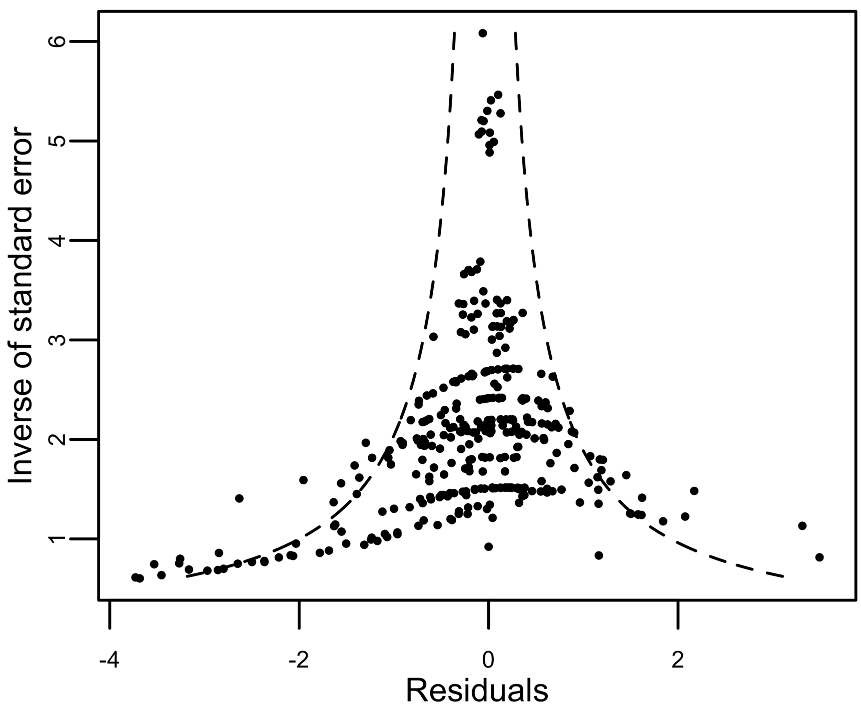

Fig S3. Funnel plot showing model residuals vs precision (inverse sampling variance).

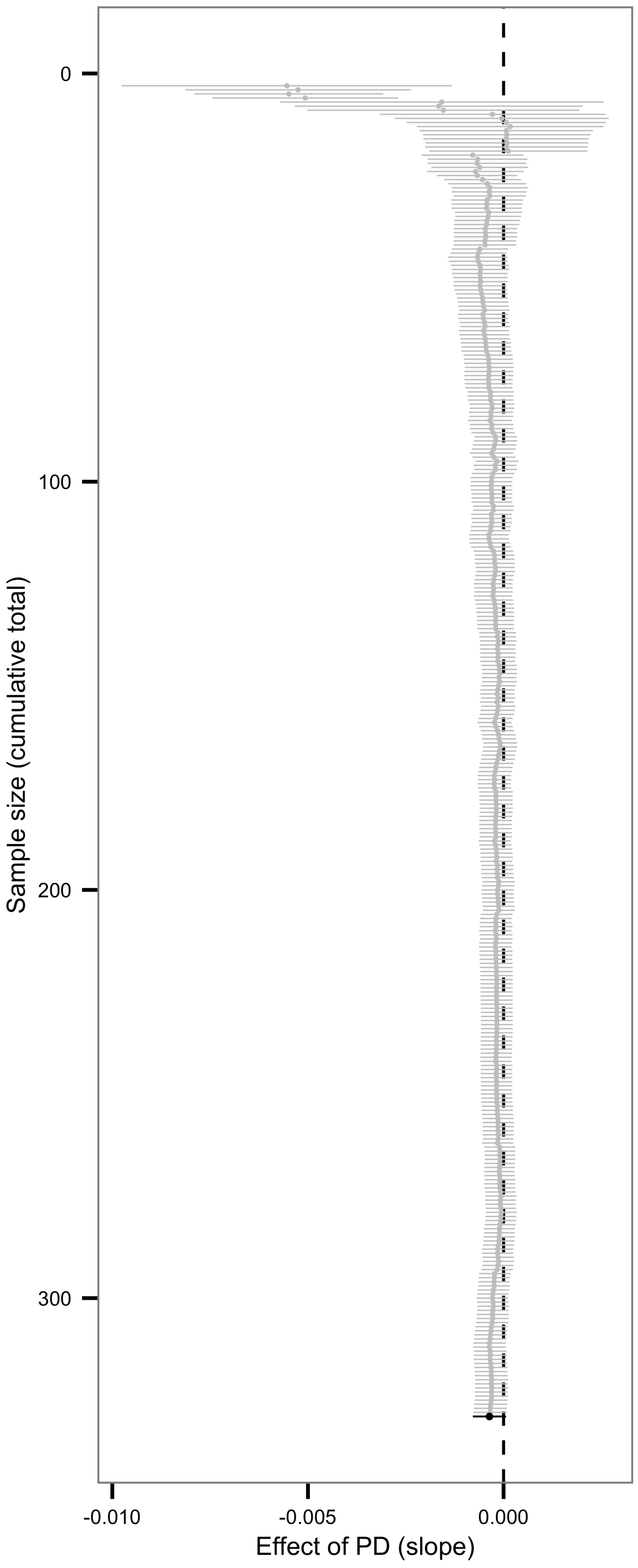

Fig S4. Forest plot of a cumulative meta-analysis of data sorted by increasing sampling variance.

Dated maximum likelihood estimate of the phylogenetic relationships between the species used in the meta-analysis:

(Pinus_sylvestris:355.000003,((Asarum_canadense:233.854429,(Cinnamomum_verum:118.800003,Virola_koschnyi:118.80000
3):115.054426):20.710305,((((Juncus_effusus:40.741861,Luzula_comosa:40.741861):72.997038,(((((Leymus_chinensis:5.60376
9,((((Aegilops_triuncialis:1.470434,Triticum_aestivum:1.470434):2.019767,(Elymus_elymoides:2.102293,Pseudoroegneria_spic
ata:2.102293):1.387909):0.793814,Agropyron_cristatum:4.284015):0.954575,Hordeum_murinum:5.238590):0.365179):3.67928
7,(Bromus_erectus:2.913028,(Bromus_tectorum:0.511798,Bromus_sterilis:0.511798):2.401230):6.370028):8.975635,((Anthoxa
nthum_odoratum:11.291884,(Arrhenatherum_elatius:7.351406,Avena_barbata:7.351406):3.940477):3.133919,((Poa_annua:9.61
3753,(Alopecurus_geniculatus:6.742626,Apera_spica-
venti:6.742626):2.871127):2.018881,(Holcus_lanatus:11.211033,(Dactylis_glomerata:9.124505,(Festuca_rubra:3.921101,(Festu
ca_idahoensis:1.238518,Festuca_ovina:1.238518):2.682583):5.203404):2.086528):0.421601):2.793169):3.832888):4.446605,Sti
pa_krylovii:22.705296):11.051309,(((Echinochloa_crus-
galli:16.700300,(Cenchrus_purpureus:12.125506,(Megathyrsus_maximus:10.236142,Panicum_capillare:10.236142):1.889365):4
.574793):0.578962,((Microstegium_vimineum:4.574397,Sorghastrum_nutans:4.574397):0.674239,(Andropogon_gerardii:1.3113
39,Schizachyrium_scoparium:1.311339):3.937297):12.030626):6.761972,(Danthonia_spicata:23.077463,(Eragrostis_pilosa:18.3
92257,(Sporobolus_heterolepis:11.622029,Hilaria_jamesii:11.622029):6.770228):4.685206):0.963771):9.715371):79.982293):8.
806196,(Prestoea_decurrens:13.523993,Iriartea_deltoidea:13.523993):109.021101):116.847923,(((Berberis_thunbergii:6.028313
,Berberis_vulgaris:6.028313):128.371690,Nigella_damascena:134.400003):0.600000,((((Rumex_occidentalis:1.288728,Rumex_
salicifolius:1.288728):36.161516,(Eriogonum_latifolium:7.110080,Eriogonum_arborescens:7.110080):30.340164):70.623823,(((
Impatiens_noli-
tangiere:20.802140,Impatiens_parviflora:20.802140):80.397862,(Northea_seychellana:53.298816,Vaccinium_corymbosum:53.2
98816):47.901186):3.608026,(((((Heracleum_mantegazzianum:2.763919,Heracleum_sphondylium:2.763919):84.681567,(Viburn
um_acerifolium:15.619121,Viburnum_dentatum:15.619121):71.826365):7.380879,((Centaurea_jacea:14.298099,Arctium_tomen
tosum:14.298099):24.685550,((Krigia_dandelion:24.412945,(Tragopogon_dubius:3.963624,Tragopogon_pratensis:3.963624):20
.449321):11.777234,((((Ratibida_columnifera:5.180634,Rudbeckia_hirta:5.180634):23.690645,(Chromolaena_odorata:19.94065
4,Lasthenia_californica:19.940654):8.930626):2.457600,(Dittrichia_viscosa:6.005039,Pulicaria_dysenterica:6.005039):25.32384
0):1.011602,(((Symphyotrichum_novae-
angliae:1.542719,Symphyotrichum_pilosum:1.542719):9.469874,((Conyza_canadensis:3.524354,(Erigeron_foliosus:3.523909,((
Erigeron_glaucus:2.808987,Erigeron_acris:2.808987):0.585645,Erigeron_annuus:3.394632):0.129277):0.000445):6.499826,(Sol
idago_canadensis:0.666282,Solidago_rugosa:0.666282):9.357898):0.988413):18.163328,((Achillea_millefolium:6.864792,Matri
caria_chamomilla:6.864792):3.929091,(Tanacetum_vulgare:8.943351,(Leucanthemum_vulgare:7.488468,(Artemisia_californica
:4.706823,((Artemisia_capillaris:3.557142,Artemisia_frigida:3.557142):0.687609,Artemisia_lavandulifolia:4.244751):0.462072)
:2.781645):1.454883):1.850532):18.382038):3.164560):3.849697):2.793470):55.842716):7.818208,(((Asclepias_tuberosa:52.76
8692,Galium_mollugo:52.768692):17.801215,(Nicotiana_tabacum:15.145322,Petunia_integrifolia:15.145322):55.424585):5.111
158,(Fraxinus_americana:54.300002,((((Salvia_lavandulifolia:3.669413,Salvia_viridis:3.669413):4.315772,(Thymus_mastichina
:0.374960,Thymus_vulgaris:0.374960):7.610225):11.620476,Galeopsis_speciosa:19.605661):4.803086,(Plantago_erecta:4.6892
77,Plantago_lanceolata:4.689277):19.719470):29.891255):21.381063):26.963508):2.163455):3.266039):8.615715,(((Epilobium_
ciliatum:111.013533,Epilobium_hirsutum:111.013533):0.000965,((Acer_rubrum:9.053574,Acer_saccharum:9.053574):88.6054
15,(Apeiba_membranacea:85.352841,(Berteroa_incana:21.515929,(Capsella_bursa-
pastoris:17.393603,Lepidium_campestre:17.393603):4.122326):63.836912):12.306149):13.355508):1.033116,((Euonymus_fortu
nei:108.812514,Viola_arvensis:108.812514):2.135315,(((Falcataria_toona:77.326307,Pentaclethra_macroloba:77.326307):0.001
301,(Lotus_heermannii:40.472457,((Vicia_cracca:1.859407,Vicia_villosa:1.859407):15.205797,(((Trifolium_repens:11.437674,(
(Trifolium_barbigerum:5.594074,Trifolium_macraei:5.594074):1.977904,(Trifolium_incarnatum:4.513454,Trifolium_pratense:4
.513454):3.058525):3.865696):1.137381,Trifolium_campestre:12.575056):2.397932,(Melilotus_albus:12.770785,Medicago_lup
ulina:12.770785):2.202203):2.092216):23.407253):36.855151):33.057681,((Quercus_ilex:12.030752,Quercus_rubra:12.030752)
:97.206891,((((Potentilla_anserina:19.378535,(Fragaria_chiloensis:1.032365,Fragaria_vesca:1.032365):18.346170):14.611685,G
eum_urbanum:33.990220):23.926438,(Prunus_ramburei:49.857547,Crataegus_monogyna:49.857547):8.059111):16.808578,Col
ubrina_spinosa:74.725236):34.512407):1.147647):0.562539):1.099785):4.642168):18.310221):104.393014):15.171717):100.435
269);

Maximum likelihood estimate of the phylogenetic relationships between the species used in the meta-analysis:

(Pinus_sylvestris:0.32213026542687833720,((Asarum_canadense:0.12977924918884586880,(Cinnamomum_verum:0.1062820
8641283407032,Virola_koschnyi:0.09253775929944123291):0.00222575607904970142):0.00782535848361852818,((((Juncus
_effusus:0.06586515227387866600,Luzula_comosa:0.14556941143005497352):0.21297147990144668639,(((((Leymus_chinen
sis:0.00526753078346379761,((((Aegilops_triuncialis:0.00584827736168993736,Triticum_aestivum:0.00244035015143775902
):0.00698979284609705428,(Elymus_elymoides:0.00612400283362341327,Pseudoroegneria_spicata:0.00157436582838840137
):0.00211657523670111865):0.00224293221731844666,Agropyron_cristatum:0.00649230899988716137):0.002100662897142
81066,Hordeum_murinum:0.01346214466906143874):0.00095738420259035696):0.00533201479708476228,(Bromus_erectus:
0.00414025361620720418,(Bromus_tectorum:0.00000121759078656250,Bromus_sterilis:0.00277839703847255299):0.007801
80317241016552):0.01591920173246611642):0.01622324014374460985,((Anthoxanthum_odoratum:0.0350911657415856753
6,(Arrhenatherum_elatius:0.01650086165773596153,Avena_barbata:0.01781057305203816860):0.00921475997599908309):0.0
0967168854313531917,((Poa_annua:0.03183408122328515444,(Alopecurus_geniculatus:0.01197588456533552233,Apera_spi
ca-
venti:0.01342410173893746408):0.00425794467309277558):0.00495763757585125728,(Holcus_lanatus:0.0230384313561666
2555,(Dactylis_glomerata:0.03174220400162158090,(Festuca_rubra:0.01141441338879397444,(Festuca_idahoensis:0.0027305
0261805742045,Festuca_ovina:0.00253300083107704582):0.00498928188654905978):0.01233855538535651164):0.00712813

979136584560):0.00129973770434853547):0.00886356521068877926):0.01490069106130058850):0.01527017092482388198,
Stipa_krylovii:0.01776184205868321792):0.02379330408604292402,(((Echinochloa_crus-
galli:0.02489133942686000411,(Cenchrus_purpureus:0.02872582884821733157,(Megathyrsus_maximus:0.02064164195721852890,Panicum_capillare:0.02017718414854716494):0.00363250913541478433):0.01009296364627991840):0.00095966279653845207,((Microstegium_vimineum:0.01293676843344941825,Sorghastrum_nutans:0.00739084986837351514):0.00157988037679002396,(Andropogon_gerardii:0.00218322586450078130,Schizachyrium_scoparium:0.00279728202210897687):0.00675512864622056577):0.02368372141913311843):0.01057262791745271387,(Danthonia_spicata:0.05757949495051500194,(Eragrostis_pilosa:0.05116209215386953446,(Sporobolus_heterolepis:0.02554981359771279353,Hilaria_jamesii:0.03422184251822954493):0.01877127201699509834):0.01486489848509208941):0.00320411600466089137):0.02613549823833080654):0.21600417636069910166):0.02818751848388810141,(Prestoea_decurrens:0.00966177064300136404,Iriartea_deltoidea:0.00992928456333385528):0.06590996608008292679):0.09566088147122847607,(((Berberis_thunbergii:0.01006741846832449015,Berberis_vulgaris:0.00301906612664218192):0.13355985815828849694,Nigella_damascena:0.13553454074539725283):0.04404175987723878832,((((Rumex_occidentalis:0.00517703459295458177,Rumex_salicifolius:0.00000121759078656250):0.07632544873607428892,(Eriogonum_latifolium:0.02140202139950753493,Eriogonum_arborescens:0.00638477630436974034):0.06108981478707040808):0.15521597515138549350,(((Impatiens_noli-
tangiere:0.03463034214709165975,Impatiens_parviflora:0.03804839327588512010):0.14245285209005612015,(Northea_seychellana:0.05158891265134109977,Vaccinium_corymbosum:0.15701904258481777510):0.00651228315040650822):0.02994923340572357845,((((Heracleum_mantegazzianum:0.00427201702770924497,Heracleum_sphondylium:0.00373497880178923431):0.12846305834988860917,(Viburnum_acerifolium:0.01784179328690416871,Viburnum_dentatum:0.00729244851824300665):0.04638587398748107021):0.00475667148885318154,((Centaurea_jacea:0.01572566019636864251,Arctium_tomentosum:0.01711731446063117709):0.02308405922514601694,((Krigia_dandelion:0.03840180959631080138,(Tragopogon_dubius:0.00443410162609179665,Tragopogon_pratensis:0.00731234614942662640):0.02909838841551821292):0.01709199946977944806,((((Ratibida_columnifera:0.01155571374285673143,Rudbeckia_hirta:0.00411613347777488835):0.03101486287808044931,(Chromolaena_odorata:0.05949347056187207633,Lasthenia_californica:0.04239407225368479332):0.02748599404708530758):0.00618167354472999673,(Dittrichia_viscosa:0.01010980854636265268,Pulicaria_dysenterica:0.00740155157547509653):0.03265891750747551558):0.00209787739865353916,(((Symphyotrichum_novae-
angliae:0.00248263582855599504,Symphyotrichum_pilosum:0.00173576092040357718):0.01005534086599297293,((Conyza_canadensis:0.00421351706832305637,(Erigeron_foliosus:0.01018128261578297999,((Erigeron_glaucus:0.00787651879389207436,Erigeron_acris:0.00442574831872430147):0.00145987386830988991,Erigeron_annuus:0.00779935570796799673):0.00037525140792042270):0.00000121759078656250):0.02116456747964482604,(Solidago_canadensis:0.00156573101018169045,Solidago_rugosa:0.00032246088300514811):0.01042379721708582181):0.00255059292935409625):0.03494356168538760576,((Achillea_millefolium:0.00830567188825046682,Matricaria_chamomilla:0.00643110107968558001):0.00221062803092605172,(Tanacetum_vulgare:0.00682594783189597286,(Leucanthemum_vulgare:0.01445392519691918350,(Artemisia_californica:0.01143397989749915002,((Artemisia_capillaris:0.01096394088332167463,Artemisia_frigida:0.00897001308635706970):0.00249472005192517311,Artemisia_lavandulifolia:0.00709164882528268582):0.00154978520569426753):0.01071814834402735547):0.00566094052999954571):0.00532098133286272763):0.03240068792055634389):0.00625371785939216225):0.00895933558367596923):0.00598142606115550056):0.08980567719190502007):0.01549380728207626866,(((Asclepias_tuberosa:0.14785231231852682954,Galium_mollugo:0.19224317452161149289):0.03879481859623966356,(Nicotiana_tabacum:0.01871665999976920500,Petunia_integrifolia:0.03121610865610638386):0.07829791595348124800):0.00223798113437042407,(Fraxinus_americana:0.10619396880398325467,((((Salvia_
lavandulifolia:0.00995112919896242246,Salvia_viridis:0.01580674650968375353):0.01752072476565291054,(Thymus_mastichina:0.00000121759078656250,Thymus_vulgaris:0.00226152573367727292):0.02367305158845313742):0.04901971349840823777,Galeopsis_speciosa:0.07738321144354361192):0.02489366139990721941,(Plantago_erecta:0.03133953620881742286,Plantago_lanceolata:0.01989373279157692284):0.15472494211856788193):0.03864305293609453240):0.03995876159265958349):0.03127775041134420869):0.00996282847692414771):0.01658307713452714105):0.00670314581176742127,(((Epilobium_ciliatum:0.19784067141940361867,Epilobium_hirsutum:0.19843790439690808802):0.00000121759078656250,((Acer_rubrum:0.01204990955628624898,Acer_saccharum:0.01410805316704323055):0.12132520028883241048,(Apeiba_membranacea:0.09371268372945443237,(Berteroa_incana:0.05406273453960236397,(Capsella_bursa-
pastoris:0.05308994425691842406,Lepidium_campestre:0.02452313587576922249):0.00992817085114541288):0.18186599245491508192):0.01170026422741633765):0.00964779426079350952):0.01375474494090014356,((Euonymus_fortunei:0.14762068778740003450,Viola_arvensis:0.20354780971984476334):0.01858317722666714272,(((Falcataria_toona:0.15070839398308125134,Pentaclethra_macroloba:0.06349209283492236733):0.00000121759078656250,(Lotus_heermannii:0.08718496972463185557,((Vicia_cracca:0.00335913640009771189,Vicia_villosa:0.00402881742835618176):0.03025394594397185985,(((Trifolium_repens:0.01597959368532626476,((Trifolium_barbigerum:0.01240175997640783484,Trifolium_macraei:0.01528879295002154651):0.00554070167174680085,(Trifolium_incarnatum:0.00787687711949547516,Trifolium_pratense:0.01312419018731169652):0.00770740316659787768):0.01227249635864337274):0.00283226887719888534,Trifolium_campestre:0.02881535259290526549):0.00648441117259541608,(Melilotus_albus:0.01945699329626629850,Medicago_lupulina:0.02676902996887487937):0.00364688209468837015):0.00489211821347563820):0.05438031693535800432):0.09065159030589445277):0.06691658971707929726,((Quercus_ilex:0.02121509266570396207,Quercus_rubra:0.00972811568300206361):0.11614727691895858352,((((Potentilla_anserina:0.03991122109523816130,(Fragaria_chiloensis:0.00140779171299708458,Fragaria_vesca:0.00189504401685503779):0.02773006027370326920):0.02705509239814734429,Geum_urbanum:0.06562137405034371151):0.04880502759925360590,(Prunus_ramburei:0.05917347306037082366,Crataegus_monogyna:0.08066819090647728285):0.01082514393903179564):0.02962739479638018189,Colubrina_spinosa:0.14384695176590930887):0.01840986144625579926):0.00251096089310159833):0.01307240038453201698):0.00698079014232880039):0.02731669681736727251):0.05203763978165305143):
0.03891278893992729249):0.00000121759078656250):0.35847901717545627154);
