# Supplementary material for: Relatedness is a poor predictor of negative plant–soil feedbacks
Source: New Phytol. 2014 Dec 31;205(3):1071–5. doi: 10.1111/nph.13238 (PMC4303931; doi:10.1111/nph.13238)
Supplement: Notes S1 — PRISMA statement. [file nph0205-1071-sd3.pdf]

Supporting Information Notes S1

PRISMA statement

PRISMA (Preferred Reporting Items for Systematic Reviews and Meta-Analyses) statement

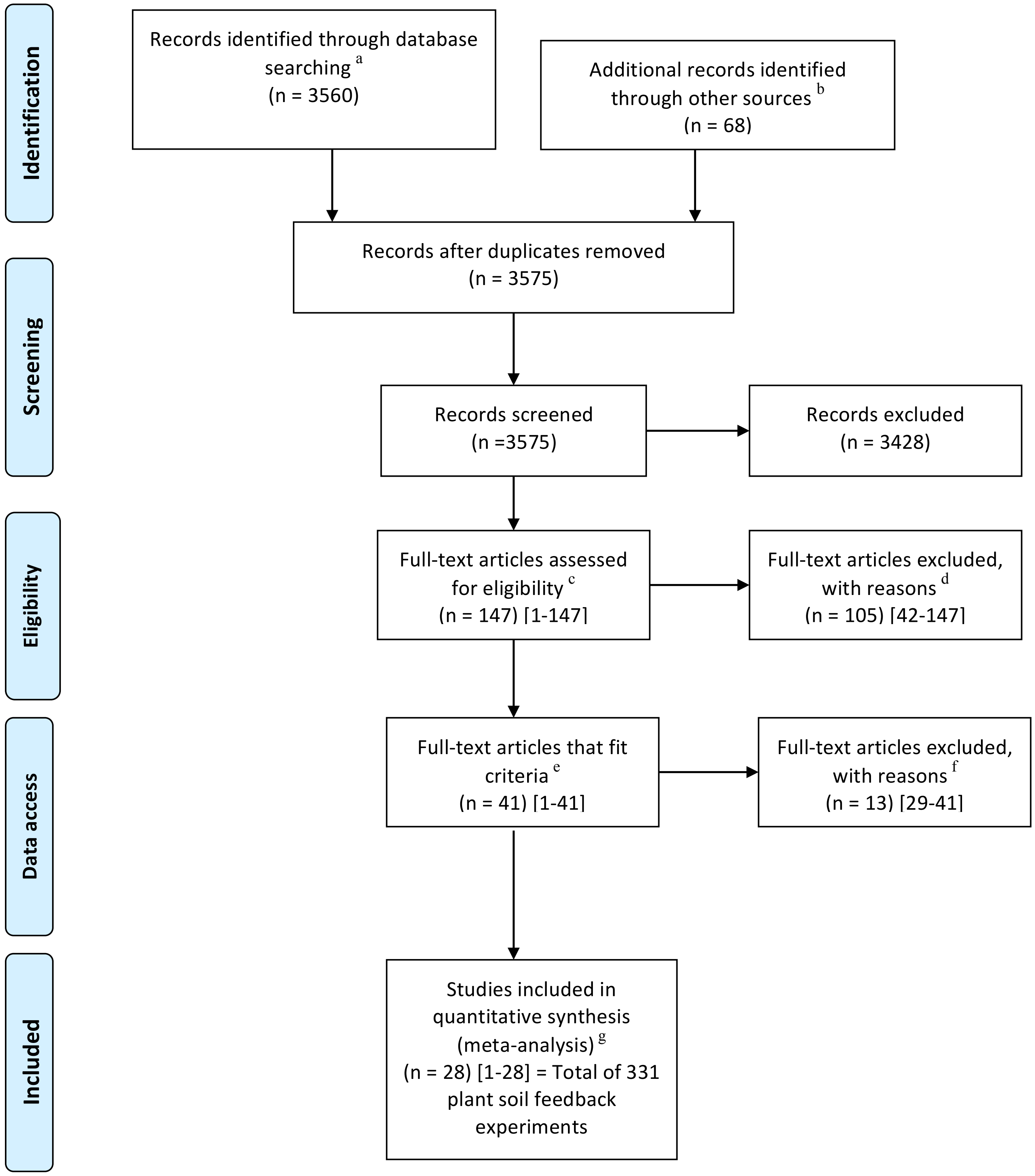

Footnotes

[a] All published work on ISI Web of Science for the last 20 years (January 1993- May 2013) were searched using 3 topic-level searches: soil* feedback* phylogeny; soil* feedback* experiment*; and plant* feedback* soil*.

[b] All plant soil feedback reviews published in the last 10 years were downloaded and references screened. Principle investigators of all main plant-soil feedback labs around the world were personally emailed and asked to contribute unpublished work or work in press.

[c] The eligibility study selection criterion was that data present the response of species grown in monoculture on soil cultured from a conspecific versus soil cultured from a heterospecific species. The response on each soil can be biomass, survival, growth or germination. [d] Any studies that did not fit this criteria were dismissed, except one study [28] which presented responses of species grown on soils cultured by its own plant family versus soil cultured by a different plant family.

[e] If data on means and SD that was readily accessible from figures this was retrieved. If data was not readily accessible authors were contacted directly to contribute their raw data. Data collection was coded to include information on plant-soil interactions between each specific plant species pair.

[f] 12 studies authors did not respond, or responded but in the end did not share the data. One study [30] was dismissed because it did not include soil culturing, but leaf litter based feedbacks.

[g] At this stage it was decided to focus only on biomass responses due to lack of survivorship, germination or growth data.

References

1. **Bever JD. 2012.** Feedback between plants and their soils in an old field community. *Ecology* **75:** 1965 –1977.

2. **Batten, KM, Scow, KM, Espeland EK. 2008**. Soil microbial community associated with an invasive grass differentially impacts native plant performance. *Microbial Ecology* **55:** 220–8.

3. **Belnap J, Phillips SL, Sherrod SK, Moldenke A. 2005.** Soil biota can change after exotic plant invasion: does this affect ecosystem processes? *Ecology* **86:** 3007–3017.

4. **Bonanomi G, Mazzoleni S. 2005.** Soil history affects plant growth and competitive ability in herbaceous species. *Community Ecology.* **6**, 23-28.

5. **Callaway RM, Thelen GC, Rodriguez A, Holben WE. 2004.** Soil biota and exotic plant invasion. *Nature* **427:** 731–3.

6. **Casper, B. B. & Castelli, J. P. 2007.** Evaluating plant-soil feedback together with competition in a serpentine grassland. *Ecology Letters* **10:** 394–400.

7. **Ehlers, B. K. & Thompson, J. 2004**. Do co-occurring plant species adapt to one another? The response of Bromus erectus to the presence of different Thymus vulgaris chemotypes. *Oecologia* **141:** 511–8.

8. **Gustafson, D. & Casper, B. 2004**. Nutrient addition affects AM fungal performance and expression of plant/fungal feedback in three serpentine grasses. *Plant Soil* **259:** 9–17.

9. **Kardol, P. & Cornips, N. 2007**. Microbe-mediated plant-soil feedback causes historical contingency effects in plant community assembly. *Ecology* **77:** 147–162.

10. **Puerta-Piñero, C., Gómez, J. M. & Zamora, R. 2006.** Species-specific effects on topsoil development affect Quercus ilex seedling performance. *Acta Oecologica* **29:** 65–71.

11. **Van Grunsven, R. H. A. *et al.* 2007.** Reduced plant?soil feedback of plant species expanding their range as compared to natives. *Journal of Ecology* **95:** 1050–1057.

12. **Casper, B. B. 2008.** Plant-soil feedback: testing the generality with the same grasses in serpentine and prairie soils *Ecology* **89:** 2154–2164.

13. **Dostál, P. & Palečková, M. 2010.** Does relatedness of natives used for soil conditioning influence plant-soil feedback of exotics? *Biological Invasions* **13:** 331–340.

14. **Elgersma, K. J., Yu, S., Vor, T. & Ehrenfeld, J. G. 2012.** Microbial-mediated feedbacks of leaf litter on invasive plant growth and interspecific competition. *Plant Soil* **356:** 341–355.

15. **Hendriks, M. *et al.* 2013.** Independent variations of plant and soil mixtures reveal soil feedback effects on plant community overyielding. *Journal of Ecology.* **101:** 287–297.

16. **Jiang, L., Han, X., Zhang, G. & Kardol, P. 2010.** The role of plant–soil feedbacks and land-use legacies in restoration of a temperate steppe in northern China. *Ecological Research* **25:** 1101–1111.

17. **Kueffer, C. 2009.** Reduced risk for positive soil-feedback on seedling regeneration by invasive trees on a very nutrient-poor soil in Seychelles. *Biological Invasions* **12:** 97–102.

18. **Perkins, L. B. & Nowak, R. S. 2012.** Soil conditioning and plant–soil feedbacks affect competitive relationships between native and invasive grasses. *Plant Ecology* **213:** 1337–1344.

19. **Smith, L. M. & Reynolds, H. L. 2012.** Positive plant-soil feedback may drive dominance of a woodland invader, Euonymus fortunei. *Plant Ecology* **213:** 853–860.

20. **Te Beest, M., Stevens, N., Olff, H. & van der Putten, W. H. 2009.** Plant-soil feedback induces shifts in biomass allocation in the invasive plant Chromolaena odorata. *Journal of Ecology* **97:** 1281–1290.

21. **Yelenik, S. & Levine, J. 2011.** The role of plant-soil feedbacks in driving native-species recovery. *Ecology* **92:** 66–74.

22. **Burns, J. & Strauss, S. 2012.** Effects of competition on phylogenetic signal and phenotypic plasticity in plant functional traits. *Ecology* **93:** 126–137.

23. **Fitzsimons, M. S. & Miller, R. M. 2010.** The importance of soil microorganisms for maintaining diverse plant communities in tallgrass prairie. *American Journal of Botany* **97,** 1937–43.

24. **Petermann, J., Fergus, A., Turnbull, L. & Schmid, B. 2008.** Janzen-Connell effects are widespread and strong enough to maintain diversity in grasslands. *Ecology* **89:** 2399–2406.

25. **McCarthy-Neumann, S. & Kobe, R. K. 2010.** Conspecific and heterospecific plant-soil feedbacks influence survivorship and growth of temperate tree seedlings. *Journal of Ecology* **98:** 408–418.

26. **McCarthy-Neumann, S. & Kobe, R. K. 2010**. Conspecific plant – soil feedbacks reduce survivorship and growth of tropical tree seedlings. *Journal of Ecology* **98:** 396–407.

27. **Pendergast, T., Burke, D. & Carson, W. 2013.** Belowground biotic complexity drives aboveground dynamics: a test of the soil community feedback model. *New Phytologist.* **197**: 1300–1310.

28. **Mehrabi, Z. 2011.** Close relatives don't make bad nieghbours: plant soil feedbacks and the dynamics of plant communities. Undergradute Thesis. University of Oxford.

29. **Perkins, L. B. & Nowak, R. S. 2013**. Native and non-native grasses generate common types of plant-soil feedbacks by altering soil nutrients and microbial communities. *Oikos* **122:** 199–208.

30. **Coq, S., Weigel, J., Bonal, D. & Hättenschwiler, S. 2012.** Litter mixture effects on tropical tree seedling growth--a greenhouse experiment. *Plant Biology.* **14:** 630–40.

31. **Reinhart, K. O., Johnson, D. & Clay, K. 2012.** Conspecific plant-soil feedbacks of temperate tree species in the southern Appalachians, USA. *PLoS One* **7,** e40680.

32. **Reinhart, K. 2012.** The organization of plant communities: negative plant-soil feedbacks and semiarid grasslands. *Ecology* **93:** 2377–2385.

33. **Liu, X. *et al.* 2012.** Experimental evidence for a phylogenetic Janzen-Connell effect in a subtropical forest. *Ecology Letters* **15:** 111–8.

34. **MacDougall, A. S., Rillig, M. C. & Klironomos, J. N. 2011.** Weak conspecific feedbacks and exotic dominance in a species-rich savannah. *Proceedings of the Royal Society: B* **278:** 2939–45.

35. **Voorde, T. F. J. Van De, Ruijten, M., Putten, W. H. Van Der & Bezemer, T. M. 2012.** Can the negative plant – soil feedback of Jacobaea vulgaris be explained by autotoxicity? *Journal of Ecology* **13:** 533–541.

36. **Schnitzer, S. & Klironomos, J. 2011.** Soil microbes drive the classic plant diversity-productivity pattern. *Ecology* **92:** 296–303.

37. **Mangan, S. A. *et al.* 2010.** Negative plant – soil feedback predicts tree-species relative abundance in a tropical forest. *Nature* **466:** 752–755.

38. **Diez, J. M. *et al.* 2010.** Negative soil feedbacks accumulate over time for non-native plant species. *Ecology Letters* **13:** 803–9.

39. **La Peña, E. *et al.* 2010.** Plant-soil feedback as a mechanism of invasion by Carpobrotus edulis. *Biological Invasions* **12:** 3637–3648.

40. **Klironomos, J. N. 2002.** Feedback with soil biota contributes to plant rarity and invasiveness in communities. *Nature* **417:** 1096–1099.

41. **Bezemer, T. M. *et al.* 2006**. Plant species and functional group effects on abiotic and microbial soil properties and plant – soil feedback responses in two grasslands. *Journal of Ecology* **5:** 893–904.

42. **Agrawal AA, Kotanen PM, Mitchell CE**. **2005**. Enemy release? An experiment with congeneric plant pairs and diverse above-and belowground enemies. *Ecology* **86**: 2979–2989.

43. **Aguilera AG**. **2011**. The influence of soil community density on plant-soil feedbacks: An important unknown in plant invasion. *Ecological Modelling* **222**: 3413–3420.

44. **Andonian K, Hierro JL, Khetsuriani L, Becerra P, Janoyan G, Villarreal D, Cavieres L, Fox LR, Callaway RM**. **2011**. Range-expanding populations of a

globally introduced weed experience negative plant-soil feedbacks. *PloS one* **6**: e20117.

45. **Ashton IW, Miller a E, Bowman WD, Suding KN**. **2008**. Nitrogen preferences and plant-soil feedbacks as influenced by neighbors in the alpine tundra. *Oecologia* **156**: 625–636.

46. **Augspurger CK, Wilkinson HT**. **2007**. Host Specificity of Pathogenic Pythium Species: Implications for Tree Species Diversity. *Biotropica* **39**: 702–708.

47. **Bainard LD, Koch a. M, Gordon a. M, Klironomos JN**. **2012**. Growth response of crops to soil microbial communities from conventional monocropping and tree-based intercropping systems. *Plant and Soil* **363**: 345–356.

48. **Bartelt-Ryser J, Joshi J, Schmid B, Brandl H, Balser T**. **2005**. Soil feedbacks of plant diversity on soil microbial communities and subsequent plant growth. *Perspectives in Plant Ecology, Evolution and Systematics* **7**: 27–49.

49. **Beckstead J, Parker IM**. **2003**. INVASIVENESS OF AMMOPHILA ARENARIA : RELEASE FROM SOIL-BORNE PATHOGENS ? *Ecology* **84**: 2824–2831.

50. **Belnap J, Phillips SL, Sherrod SK, Moldenke A**. **2005**. Soil biota can change after exotic plant invasion: does this affect ecosystem processes? *Ecology* **86**: 3007–3017.

51. **Bever JD, Broadhurst LM, Thrall PH**. **2013**. Microbial phylotype composition and diversity predicts plant productivity and plant-soil feedbacks. *Ecology letters* **16**: 167–174.

52. **Bever JD, Dickie I a, Facelli E, Facelli JM, Klironomos J, Moora M, Rillig MC, Stock WD, Tibbett M, Zobel M**. **2010**. Rooting theories of plant community ecology in microbial interactions. *Trends in ecology & evolution* **25**: 468–478.

53. **Bever JD, Platt TG, Morton ER**. **2012**. Microbial population and community dynamics on plant roots and their feedbacks on plant communities. *Annual review of microbiology* **66**: 265–283.

54. **Bever JD, Westover KM, Antonovics J**. **1997**. incorporating the soil community into plant populaiton dynamics: the utility of the feedback approach. *Journal of Ecology*.

55. **Bezemer TM, Harvey J a, Kowalchuk G a, Korpershoek H, van der Putten WH**. **2006**. Interplay between Senecio jacobaea and plant, soil, and aboveground insect community composition. *Ecology* **87**: 2002–2013.

56. **Bezemer T, van der Putten WH, Martens H, van de Voorde TFJ, Mulder PPJ, Kostenko O**. **2013**. Above- and below-ground herbivory effects on below-

ground plant-fungus interactions and plant-soil feedback responses (M Hutchings, Ed.). *Journal of Ecology* **101**: 325–333.

57. **Blank RR, Sforza R**. **2007**. Plant–soil relationships of the invasive annual grass Taeniatherum caput-medusae: a reciprocal transplant experiment. *Plant and Soil* **298**: 7–19.

58. **Bodelier PLE, Stomp M, Santamaria L, Klaassen M, Laanbroek HJ**. **2006**. Animal-plant-microbe interactions: direct and indirect effects of swan foraging behaviour modulate methane cycling in temperate shallow wetlands. *Oecologia* **149**: 233–244.

59. **Bonanomi G, Rietkerk M, Dekker SC, Mazzoleni S**. **2008**. Islands of fertility induce co-occurring negative and positive plant-soil feedbacks promoting coexistence. *Plant Ecology*.

60. **Brandt AJ, de Kroon H, Reynolds HL, Burns JH**. **2013**. Soil heterogeneity generated by plant-soil feedbacks has implications for species recruitment and coexistence (W van der Putten, Ed.). *Journal of Ecology* **101**: 277–286.

61. **Brandt AJ, Seabloom EW, Hosseini PR**. **2009**. Phylogeny and provenance affect plant-soil feedbacks in invaded California grasslands. *Ecology* **90**: 1063–1072.

62. **Buetof A, Bruelheide H**. **2011**. Effects of an unspecialized soil pathogen on congeneric plant species with different geographic distributions. *Preslia*: 205–217.

63. **BURNS AND Strauss SY**. **2011**. More closely related species are more ecologically similar in an experimental test. *Proceedings of the National Academy of Sciences* **109**: 3599.

64. **Callaway RM**. **2000**. Invasive Plants Versus Their New and Old Neighbors: A Mechanism for Exotic Invasion. *Science* **290**: 521–523.

65. **Callaway RM, Thelen GC, Barth S**. **2004**. Soil fungi alter interactions between the invader Centaurea maculosa and North American natives. *Ecology* **85**: 1062–1071.

66. Der Stoel, C. D. Van. Development of a negative plant–soil feedback in the expansion zone of the clonal grass Ammophila arenaria following root formation and nematode. *J.Ecol* (2002).

67. **Deyn GB De**. **2004**. Plant community development is affected by nutrients and soil biota. *Journal of …*: 824–834.

68. **Dickie I a., Schnitzer S a., Reich PB, Hobbie SE**. **2007**. Is oak establishment in old-fields and savanna openings context dependent? *Journal of Ecology* **95**: 309–320.

69. **Ehrenfeld JG, Ravit B, Elgersma K**. **2005**. Feedback in the Plant-Soil System. *Annual Review of Environment and Resources* **30**: 75–115.

70. **Felker-Quinn E, Bailey JK, Schweitzer JA**. **2011**. Soil biota drive expression of genetic variation and development of population-specific feedbacks in an invasive plant EMMI. *Ecology* **92**: 1208–1214.

71. **Flory SL, Clay K**. **2013**. Pathogen accumulation and long-term dynamics of plant invasions (P Thrall, Ed.). *Journal of Ecology* **101**: 607–613.

72. **Gillespie IG, Allen EB**. **2006**. Effects of soil and mycorrhizae from native and invaded vegetation on a rare California forb. *Applied Soil Ecology* **32**: 6–12.

73. **Grman E, Suding KN**. **2010**. Within-Year Soil Legacies Contribute to Strong Priority Effects of Exotics on Native California Grassland Communities. *Restoration Ecology* **18**: 664–670.

74. **Harrison K a., Bardgett RD**. **2010**. Influence of plant species and soil conditions on plant-soil feedback in mixed grassland communities. *Journal of Ecology* **98**: 384–395.

75. **Heijden MG a., Wagg C**. **2012**. Soil microbial diversity and agro-ecosystem functioning. *Plant and Soil* **363**: 1–5.

76. **Holah JC, Alexander HM**. **1999**. Soil pathogenic fungi have the potential to affect the co- existence of two tallgrass prairie species. *Journal of Ecology*: 598–608.

77. **Huang L-F, Song L-X, Xia X-J, Mao W-H, Shi K, Zhou Y-H, Yu J-Q**. **2013**. Plant-soil feedbacks and soil sickness: from mechanisms to application in agriculture. *Journal of chemical ecology* **39**: 232–242.

78. **Inderjit, van der Putten WH**. **2010**. Impacts of soil microbial communities on exotic plant invasions. *Trends in ecology & evolution* **25**: 512–519.

79. **Johnson DJ, Beaulieu WT, Bever JD, Clay K**. **2012**. Conspecific negative density dependence and forest diversity. *Science (New York, N.Y.)* **336**: 904–907.

80. **Jordan NR, Larson DL, Huerd SC**. **2007**. Soil modification by invasive plants: effects on native and invasive species of mixed-grass prairies. *Biological Invasions* **10**: 177–190.

81. **Kardol P, Bezemer TM, van der Putten WH**. **2006**. Temporal variation in plant-soil feedback controls succession. *Ecology letters* **9**: 1080–1088.

82. **Kardol P, De Deyn GB, Laliberté E, Mariotte P, Hawkes C V**. **2013**. Biotic plant-soil feedbacks across temporal scales (W van der Putten, Ed.). *Journal of Ecology* **101**: 309–315.

83. **Knevel IC, Lans T, Menting FBJ, Hertling UM, van der Putten WH**. **2004**. Release from native root herbivores and biotic resistance by soil pathogens in a new habitat both affect the alien Ammophila arenaria in South Africa. *Oecologia* **141**: 502–510.

84. **Kotanen PM**. **2007**. Effects of fungal seed pathogens under conspecific and heterospecific trees in a temperate forest. *Canadian Journal of Botany* **85**: 918–925.

85. **Kulmatiski A, Beard KH**. **2011**. Long-term plant growth legacies overwhelm short-term plant growth effects on soil microbial community structure. *Soil Biology and Biochemistry* **43**: 823–830.

86. **Kulmatiski A, Beard KH, Heavilin J**. **2012**. Plant-soil feedbacks provide an additional explanation for diversity-productivity relationships. *Proceedings. Biological sciences / The Royal Society* **279**: 3020–3026.

87. **Kulmatiski A, Beard KH, Stark JM**. **2006**. Soil history as a primary control on plant invasion in abandoned agricultural fields. *Journal of Applied Ecology* **43**: 868–876.

88. **Kulmatiski A, Beard KH, Stevens JR, Cobbold SM**. **2008**. Plant-soil feedbacks: a meta-analytical review. *Ecology letters* **11**: 980–992.

89. **Kulmatiski A, Heavilin J, Beard KH**. **2011**. Testing predictions of a three-species plant-soil feedback model. *Journal of Ecology*: no–no.

90. **Lankau R a, Strauss SY**. **2007**. Mutual feedbacks maintain both genetic and species diversity in a plant community. *Science (New York, N.Y.)* **317**: 1561–1563.

91. **Lankau R a., Wheeler E, Bennett AE, Strauss SY**. **2011**. Plant-soil feedbacks contribute to an intransitive competitive network that promotes both genetic and species diversity. *Journal of Ecology* **99**: 176–185.

92. **Lee MR, Flory SL, Phillips RP**. **2012**. Positive feedbacks to growth of an invasive grass through alteration of nitrogen cycling. *Oecologia* **170**: 457–465.

93. **Liu Y, Yu S, Xie Z-P, Staehelin C**. **2012**. Analysis of a negative plant-soil feedback in a subtropical monsoon forest. *Journal of Ecology* **100**: 1019–1028.

94. **Mangan S a, Herre E a, Bever JD**. **2010**. Specificity between Neotropical tree seedlings and their fungal mutualists leads to plant-soil feedback. *Ecology* **91**: 2594–2603.

95. **Mangla S, Callaway RM**. **2007**. Exotic invasive plant accumulates native soil pathogens which inhibit native plants. *Journal of Ecology*: 071031082432001–???

96. **Manning P, Morrison S a, Bonkowski M, Bardgett RD**. **2008**. Nitrogen enrichment modifies plant community structure via changes to plant-soil feedback. *Oecologia* **157**: 661–673.

97. **Mariotte P, Vandenberghe C, Meugnier C, Rossi P, Bardgett RD, Buttler A**. **2013**. Perspectives in Plant Ecology , Evolution and Systematics Subordinate plant species impact on soil microbial communities and ecosystem functioning in grasslands : Findings from a removal experiment. **15**: 77–85.

98. **Maron JL, Marler M, Klironomos JN, Cleveland CC**. **2011**. Soil fungal pathogens and the relationship between plant diversity and productivity. *Ecology letters* **14**: 36–41.

99. **McCarthy-Neumann S, Ibáñez I**. **2012**. Tree range expansion may be enhanced by escape from negative plant-soil feedbacks. *Ecology* **93**: 2637–2649.

100. **McCarthy-Neumann S, Ibáñez I**. **2013**. Plant–soil feedback links negative distance dependence and light gradient partitioning during seedling establishment. *Ecology* **94**: 780–786.

101. **McCarthy-Neumann S, Kobe RK**. **2008**. Tolerance of soil pathogens co-varies with shade tolerance across species of tropical tree seedlings. *Ecology* **89**: 1883–1892.

102. **Meijer SS, Holmgren M, Van der Putten WH**. **2011**. Effects of plant-soil feedback on tree seedling growth under arid conditions. *Journal of Plant Ecology* **4**: 193–200.

103. **Meiman PJ, Redente EF, Paschke MW**. **2006**. The role of the native soil community in the invasion ecology of spotted (Centaurea maculosa auct. non Lam.) and diffuse (Centaurea diffusa Lam.) knapweed. *Applied Soil Ecology* **32**: 77–88.

104. **Middleton EL, Bever JD**. **2012**. Inoculation with a Native Soil Community Advances Succession in a Grassland Restoration. *Restoration Ecology* **20**: 218–226.

105. **Mommer L, Van Ruijven J, De Caluwe H, Smit-Tiekstra AE, a.M. Wagemaker C, Joop Ouborg N, Bögemann GM, Van Der Weerden GM, Berendse F, De Kroon H**. **2010**. Unveiling below-ground species abundance in a biodiversity experiment: a test of vertical niche differentiation among grassland species. *Journal of Ecology* **98**: 1117–1127.

106. **Mordecai E a. 2011**. Pathogen impacts on plant communities: unifying theory, concepts, and empirical work. *Ecological Monographs* **81**: 429–441.

107. **Morriën E, Engelkes T, van der Putten WH**. **2011**. Additive effects of aboveground polyphagous herbivores and soil feedback in native and range-expanding exotic plants. *Ecology* **92**: 1344–1352.

108. **Morris C, Call C a., Monaco T a., Grossl PR, Dewey S a. 2006**. Evaluation of elemental allelopathy in Acroptilon repens (L.) DC. (Russian Knapweed). *Plant and Soil* **289**: 279–288.

109. **Nijjer S, Rogers WE, Siemann E**. **2007**. Negative plant-soil feedbacks may limit persistence of an invasive tree due to rapid accumulation of soil pathogens. *Proceedings. Biological sciences / The Royal Society* **274**: 2621–2627.

110. **Niu H, Liu W, Wan F, Liu B**. **2007**. An invasive aster (Ageratina adenophora) invades and dominates forest understories in China: altered soil microbial communities facilitate the invader and inhibit natives. *Plant and Soil* **294**: 73–85.

111. **Packer a, Clay K**. **2000**. Soil pathogens and spatial patterns of seedling mortality in a temperate tree. *Nature* **404**: 278–281.

112. **Padilla FM, Mattingly WB, Swedo BL, Clay K, Reynolds HL**. **2012**. Negative plant-soil feedbacks dominate seedling competitive interactions of North American successional grassland species (S Gusewell, Ed.). *Journal of Vegetation Science* **23**: 667–676.

113. **Peltzer D a. 2001**. Plant responses to competition and soil origin across a prairie-forest boundary. *Journal of Ecology* **89**: 176–185.

114. **Pernilla Brinkman E, Van der Putten WH, Bakker E-J, Verhoeven KJF**. **2010**. Plant-soil feedback: experimental approaches, statistical analyses and ecological interpretations. *Journal of Ecology* **98**: 1063–1073.

115. **Pizano C, Mangan S a, Herre EA, Eom A-H, Dalling JW**. **2011**. Above- and belowground interactions drive habitat segregation between two cryptic species of tropical trees. *Ecology* **92**: 47–56.

116. **Ponge JF**. **2013**. Plant soil feedbacks mediated by humus forms : A review. *Soil Biology and Biochemistry* **57**: 1048–1060.

117. **Pregitzer CC, Bailey JK, Hart SC, Schweitzer J a. 2010**. Soils as agents of selection: feedbacks between plants and soils alter seedling survival and performance. *Evolutionary Ecology* **24**: 1045–1059.

118. **Reinhart KO, Callaway RM**. **2004**. Soil biota facilitate exotic Acer invasions in Europe and North America. *Ecological Applications*.

119. **Reinhart KO, Clay K**. **2009**. Spatial variation in soil-borne disease dynamics of a temperate tree, Prunus serotina. *Ecology* **90**: 2984–2993.

120. **Reinhart KO, Greene E, Callaway RM**. **2005**. Effects of Acer platanoides invasion on understory plant communities and tree regeneration in the northern Rocky Mountains. *Ecography* **5**: 573–582.

121. **Reinhart KO, Packer A**. **2003**. Plant–soil biota interactions and spatial distribution of black cherry in its native and invasive ranges. *Ecology …*: 1046–1050.

122. **Reinhart KO, Royo AA**. **2005**. Soil feedback and pathogen activity in Prunus serotina throughout its native range. *Journal of …*: 890–898.

123. **Revilla T a., Veen GF, Eppinga MB, Weissing FJ**. **2012**. Plant–soil feedbacks and the coexistence of competing plants. *Theoretical Ecology* **6**: 99–113.

124. **Reynolds HL, Packer A, Bever JD, Clay K**. **2003**. Grassroots Ecology: Plant–Microbe–Soil Interactions As Drivers of Plant Community Structure and Dynamics. *Ecology* **84**: 2281–2291.

125. **Rudgers J a., Orr S**. **2009**. Non-native grass alters growth of native tree species via leaf and soil microbes. *Journal of Ecology* **97**: 247–255.

126. **Scharfy D, Güsewell S, Gessner MO, Venterink HO**. **2010**. Invasion of Solidago gigantea in contrasting experimental plant communities: effects on soil microbes, nutrients and plant-soil feedbacks. *Journal of Ecology* **98**: 1379–1388.

127. **Schweizer D, Gilbert GS, Holl KD**. **2013**. Phylogenetic ecology applied to enrichment planting of tropical native tree species. *Forest Ecology and Management* **297**: 57–66.

128. **Shannon S, Flory SL, Reynolds H**. **2012**. Competitive context alters plant-soil feedback in an experimental woodland community. *Oecologia* **169**: 235–243.

129. **Sigüenza C, Corkidi L, Allen EB**. **2006**. Feedbacks of Soil Inoculum of Mycorrhizal Fungi Altered by N Deposition on the Growth of a Native Shrub and an Invasive Annual Grass. *Plant and Soil* **286**: 153–165.

130. **Stinson K a, Campbell S a, Powell JR, Wolfe BE, Callaway RM, Thelen GC, Hallett SG, Prati D, Klironomos JN**. **2006**. Invasive plant suppresses the growth of native tree seedlings by disrupting belowground mutualisms. *PLoS biology* **4**: e140.

131. **Suding KN, Larson JR, Thorsos E, Steltzer H, Bowman WD**. **2004**. Species effects on resource supply rates: do they influence competitive interactions? *Plant Ecology formerly `Vegetatio'* **175**: 47–58.

132. **Suding KN, Stanley Harpole W, Fukami T, Kulmatiski A, MacDougall AS, Stein C, van der Putten WH**. **2013**. Consequences of plant-soil feedbacks in invasion. *Journal of Ecology* **101**: 298–308.

133. **Suter M, Ramseier D, Connolly J, Edwards PJ**. **2010**. Species identity and negative density dependence lead to convergence in designed plant mixtures of twelve species. *Basic and Applied Ecology* **11**: 627–637.

134. **Troelstra SR, Wagenaar R**. **2001**. Interpretation of bioassays in the study of interactions between soil organisms and plants involvement of nutrient factors. *New Phytologist.*

135. **Van de Voorde TFJ**. **2012**. Soil inoculation method determines the strength of plant e soil interactions. *Soil Biology and ...* **55**: 1–6.

136. **Van de Voorde TFJ, van der Putten WH, Martijn Bezemer T**. **2011**. Intra- and interspecific plant-soil interactions, soil legacies and priority effects during old-field succession. *Journal of Ecology* **99**: 945–953.

137. **Van der Putten WH, Bardgett RD 2009.** Empirical and theoretical challenges in aboveground belowground ecology. *Oecologia*

138. **Van der Putten, WH *et al.* 2007.** Soil feedback of exotic savanna grass relates to pathogen absence and mycorrhizal selectivity. *Ecology* **88:** 978–988.

139. **Van der Putten, W. H. A. 2009.** multitrophic perspective on functioning and evolution of facilitation in plant communities. *Journal of Ecology* **97:** 1131–1138.

140. **Van der Putten WH *et al.* 2013. P**lant-soil feedbacks: the past, the present and future challenges. *Journal of Ecology* **101**, 265–276.

141. **Van der Stoel CD, van der Putten WH, Duyts H. 2002.** Development of a negative plant-soil feedback in the expansion zone of the clonal grass Ammophila arenaria following root formation and nematode colonization. *Journal of Ecology* **90:** 978–988 (2002).

142. **Van Grunsven RH, Van der Putten WH, Bezemer T, Berendse F, Veenendaal EM. 2010.** Plant-soil interactions in the expansion and native range of a poleward shifting plant species. *Global Change Biology.* **16:** 380–385.

143. **Van Grunsven RHA, van der Putten WH, Bezemer TM, Veenendaal E M. 2010.** Plant-soil feedback of native and range-expanding plant species is insensitive to temperature. *Oecologia* **162:** 1059–1069.

144. **Vinton MA, Goergen EM 2006.** Plant–Soil Feedbacks Contribute to the Persistence of Bromus inermis in Tallgrass Prairie. *Ecosystems* **9**: 967–976

145. **Vivanco L, Austin AT 2008.** Tree species identity alters forest litter decomposition through long-term plant and soil interactions in Patagonia, Argentina. *Journal of Ecology* **96:** 727–736.

146. **Wang SJ, Ruan HH**. **2008**. Feedback mechanisms of soil biota to aboveground biology in terrestrial ecosystems. *Biodiversity Science*. **4**: 4-407.

147. **Weltzin JF, McPherson GR**. **1999**. Facilitation of conspecific seedling recruitment and shifts in temperate savanna ecotones. *Ecological Monographs* **69**: 513–534.
